# Comprehensive Analysis of the Immune Infiltrates and PD-L1 of m^6^A RNA Methylation Regulators in Hepatocellular Carcinoma

**DOI:** 10.3389/fcell.2021.681745

**Published:** 2021-06-30

**Authors:** Yangtao Xu, Xiaoqin He, Junjian Deng, Lin Xiong, Yue Li, Xiaoyu Zhang, Wenliang Chen, Xin Liu, Ximing Xu

**Affiliations:** ^1^Cancer Center, Renmin Hospital of Wuhan University, Wuhan, China; ^2^Department of Pathology, Renmin Hospital of Wuhan University, Wuhan, China

**Keywords:** hepatocellular carcinoma, m^6^A RNA methylation, PD-L1, tumor immune microenvironment, immune infiltrates, prognosis

## Abstract

Recently, *N*^6^-methyladenosine (m^6^A) RNA methylation in eukaryotic mRNA has become increasingly obvious in the pathogenesis and prognosis of cancer. Moreover, tumor microenvironment is involved in the regulation of tumorigenesis. In our research, the clinical data, including 374 tumor and 50 normal patients, were obtained from The Cancer Genome Atlas (TCGA). Then 19 m^6^A regulators were selected from other studies. Hepatocellular carcinoma (HCC) patients were clustered in cluster1/2, according to the consensus clustering for the m^6^A RNA regulators. We found that m^6^A regulators were upregulated in cluster1. The cluster1 was associated with higher programmed death ligand 1 (PD-L1) expression level, higher immunoscore, worse prognosis, and distinct immune cell infiltration compared with cluster2. Five risk signatures were identified, including YTH N6-methyladenosine RNA-binding protein 1, YTHDF2, heterogeneous nuclear ribonucleoprotein C, WT1-associated protein, and methyltransferase-like 3, based on univariate Cox and least absolute shrinkage and selection operator regression analysis. High-risk group and low-risk group HCC patients were selected based on the risk score. Similarly, the high-risk group was extremely associated with higher PD-L1 expression level, higher grade, and worse overall survival (OS). Also, cluster1 was mainly enriched in high-risk group. Receiver operating characteristic (ROC) and a nomogram were used to predict the ability and the probability of 3- and 5-year OS of HCC patients. The time-dependent ROC curve (AUC) reached 0.77, 0.67, and 0.68 at 1, 3, and 5 years in the training dataset. Also, AUC areas of 1, 3, and 5 years were 0.7, 0.63, and 0.55 in the validation dataset. The gene set enrichment analysis showed that MTOR signaling pathway and WNT signaling pathway were correlated with cluster1 and high-risk group. Collectively, the research showed that the m^6^A regulators were significantly associated with tumor immune microenvironment in HCC. Risk characteristics based on m^6^A regulators may predict prognosis in patients with HCC and provide a new therapeutic target for improving the efficacy of immunotherapy.

## Introduction

Hepatocellular carcinoma (HCC) is one of the most prevalent cancers in the world and the fourth most deadly cancer (Forner et al., 2012; Bray et al., 2018). In China alone, more than 466,100 people are diagnosed with HCC, and approximately 422,100 individuals succumb to HCC (Chen et al., 2016). Behind the high incidence of HCC, there are several modifiable factors, including hepatitis virus, alcohol abuse, smoking, and metabolic syndrome. Especially the hepatitis virus, in most of Africa and Asia, such as China, hepatitis B virus is the single leading risk factor for HCC, whereas in northern Europe and the United States, hepatitis C virus is the major risk factor (El-Serag, 2012). According to the location and clinical stage of HCC, the main treatment methods include surgery, chemotherapy, and radiotherapy, but the prognosis of advanced HCC is poor and treatment methods are limited. Interestingly, immunotherapy has developed rapidly in the past few years, improving survival of patients with HCC (Zhong et al., 2021). However, only a few patients with HCC could benefit from this treatment, and most patients still respond negatively to immune therapies. Immune system imbalance is involved in the development of immune-characterized HCC. For example, activation and ability of NK cells are reduced in HCC patients. Tumor-associated neutrophils are significantly associated with the development of HCC. Postoperative HCC patients with high-level lymphocyte infiltration, especially T cells, are closely related to better prognosis (Unitt et al., 2006; Margetts et al., 2018; Fu et al., 2019). Therefore, to identify more biomarkers for accurate prediction of prognosis and to optimize individualized immunotherapy management to a large extent, the mechanism of tumor immune microenvironment (TIME) needs to be further explored.

Post-transcriptional modification is also involved in the progression of various diseases and has attracted significant attention in the biomedicine (He et al., 2019). *N*^6^-methyladenosine (m^6^A) is the methylated modification of the sixth N atom of adenine and the most abundant mRNA modification among numerous RNA modifications. The average 1,000 nt contain one or two m^6^A residues (Krug et al., 1976; Du et al., 2019). There are three types in the m^6^A regulators, including writers, erasers, and readers. The m^6^A is catalyzed by the methyltransferase complex (MTC), also known as the “writer,” which included methyltransferase-like 3 (METTL3), METTL14, METTL16, WT1-associated protein (WTAP), zinc finger CCCH domain-containing protein 13 (ZC3H13), ZCCHC4, KIAA1429, zinc finger protein (ZFP217), RNA-binding motif protein 15 (RBM15), and RBM15B (Ping et al., 2014; Patil et al., 2016; Wang et al., 2016, 2017; Warda et al., 2017; Knuckles et al., 2018; Yue et al., 2018; Song et al., 2019; Pinto et al., 2020). Demethylase, also termed as “eraser,” comprising fat mass- and obesity-associated protein (FTO) and α-ketoglutarate-dependent dioxygenase alkB homolog 5 (ALKBH5), removes m^6^A methylation groups from RNA (Zhao et al., 2014; Zhang et al., 2017). The “readers” bind to the m^6^A methylation site, which include YTH domain-containing 1 (YTHDC1), YTHDC2, heterogeneous nuclear ribonucleoprotein C (HNRNPC), HNRNPA2B1 YTH N6-methyladenosine RNA-binding protein 1 (YTHDF1), YTHDF2, and YTHDF3 (Wang et al., 2014, 2015; Alarcón et al., 2015; Liu et al., 2015; Hsu et al., 2017; Shi et al., 2017; Kasowitz et al., 2018). In HCC, patients with higher levels of YTHDF1 and METTL3 are associated with worse overall survival (OS). YTHDF1 can mediate the m^6^A to enhance Snail expression. METTL3 deficiency leads to a decrease in m^6^A, which blocks the EMT of cancer cells (Lin et al., 2019; Zhou et al., 2019). However, the mechanisms of other m^6^A methylation regulators in liver cancer remain unclear. Moreover, the correlation between m^6^A methylation modulator and programmed death ligand 1 (PD-L1) remains to be fully explored.

In this research, the relationship of m^6^A RNA methylation regulators with PD-L1, prognosis, and TIME in HCC was analyzed. In addition, we established a cluster subtype and risk model for m^6^A regulators to identify novel HCC markers and novel therapeutic strategies (Supplementary Figure 1).

## Materials and Methods

### Dataset Source

The HCC clinical data and the mRNA expression data of patients were obtained from The Cancer Genome Atlas (TCGA) data portal.^1^ The research included 374 tumor and 50 normal samples. The TCGA data were downloaded by using the R package “TCGAbiolinks” (Colaprico et al., 2016).

### Identification of Consensus Clustering and Prognosis for m^6^A RNA Methylation Regulators

In the research, 19 m^6^A regulators were selected. HCC patients were clustered into cluster1 (*n* = 166) and cluster2 (*n* = 169) by using R package “ConsensusClusterPlus”.^2^ Furthermore, we used univariate Cox analysis and least absolute shrinkage and selection operator (LASSO) regression to identify five risk signatures, including YTHDF1, YTHDF2, HNRNPC, WTAP, and METTL3, and a risk score was generated for each HCC patient. Kaplan–Meier curves and receiver operating characteristic (ROC) curves were used to assess the prognostic capacity of the risk scores.

### Identification of the Correction Between m^6^A RNA Regulators and TIME in HCC

The R package “estimate” was used to calculate the immunoscore for each patient with the ESTIMATE algorithm. The fraction of 22 immune cell types between cluster1 and cluster2 and the gene set enrichment analysis (GSEA) were analyzed through Sangerbox website.^3^ Also, the effect of somatic copy number change (CNA) based on m^6^A modulator signal on immune cell infiltration was explored based on CIBERSORT.^4^

### A Predictive Nomogram

A nomogram was built to investigate the probability of prognosis in patients (Iasonos et al., 2008). Then the discrimination and accuracy of the nomogram were assessed by the concordance index (C-index) and a calibration.

### Statistical Analysis

Statistical tests were carried out using GraphPad Prism 8.0 (GraphPad Software, San Diego, CA, United States) and R version 4.0.2 (version 4.0.2^5^). “limma,” “ConsensusClusterPlus,” “survival,” “glmnet,” “edgeR,” and “timeROC” R package were used.

## Results

### The m^6^A RNA Methylation Regulators Expressed Differently in HCC

**FIGURE 1:**
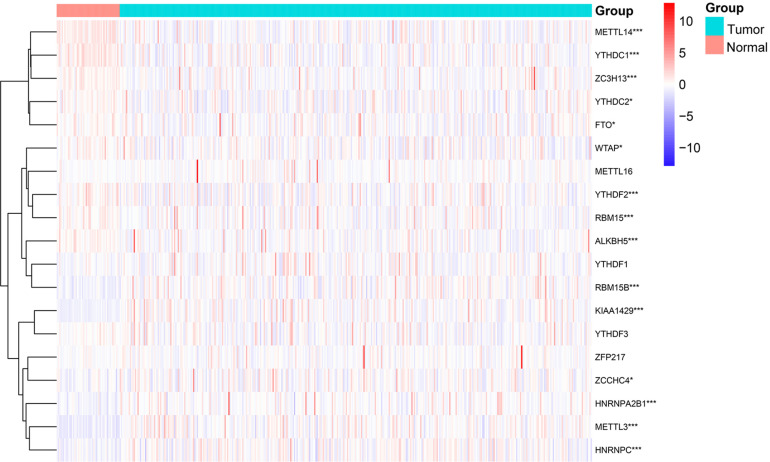
Expression levels of m^6^A RNA methylation regulators in normal and tumor samples. *p < 0.05 and ***p < 0.001.

By comparing 50 normal and 374 tumor tissues, METTL14, YTHDC1, ZC3H13, ALKBH5, YTHDF2, and RBM15 had extremely lower expression in tumor tissues (*p* < 0.001, Figure 1). Also, the expression levels of YTHDC2, WTAP, and FTO were markedly lower in tumor tissues (*p* < 0.05). On the contrary, METTL3, KIAA1429, RBM15B, HNRNPA2B1, and HNRNPC had significantly higher expression in tumor tissues (*p* < 0.001). The results showed that m^6^A regulators could be involved in biological development of HCC.

### The Consensus Cluster of m^6^A RNA Methylation Regulators Was Significantly Associated With Clinical Signatures of Patients With HCC

**FIGURE 2:**
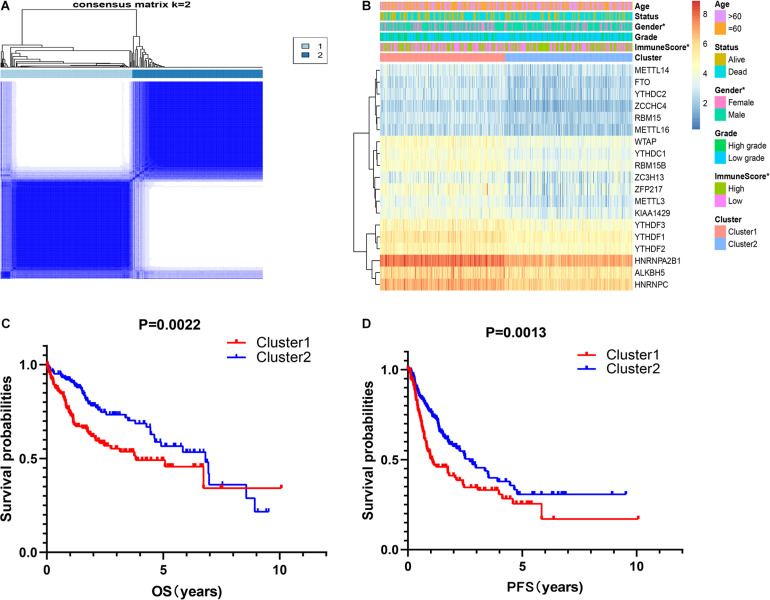
Correlation of consensus clustering for m^6^A RNA methylation regulators with the characteristics and survival of patients with HCC. **(A)** Consensus clustering matrix for *k* = 2. **(B)** Heatmap of correlation of m^6^A RNA methylation regulators with characteristics of HCC patients. **(C,D)** OS and PFS of HCC patients in cluster1 and cluster2. *p < 0.05.

It is determined that *k* = 2 has the best clustering stability from *k* = 2 to 9, based on the similarity between the expression level of m^6^A regulators and the proportion of fuzzy clustering measures (Supplementary Figure 2). According to the expression levels of the m^6^A regulators, 335 HCC patients were clustered into cluster1 and cluster2 (*n*1 = 166, *n*2 = 169, Figure 2A). The findings indicated that the expression level of individual m^6^A methylation regulators in cluster1 was higher than in cluster2 (Figure 2B). Moreover, the clinical futures were compared between cluster1 and cluster2. Female and low immunoscore HCC patients were significantly enriched in cluster1 (*p* < 0.05, Figure 2B). The OS (OS, *p* = 0.0022) and progression-free survival (PFS, *p* = 0.0013) were worse in cluster1 (Figures 2C,D).

### Correction Between PD-L1 and m^6^A RNA Methylation Regulators

**FIGURE 3:**
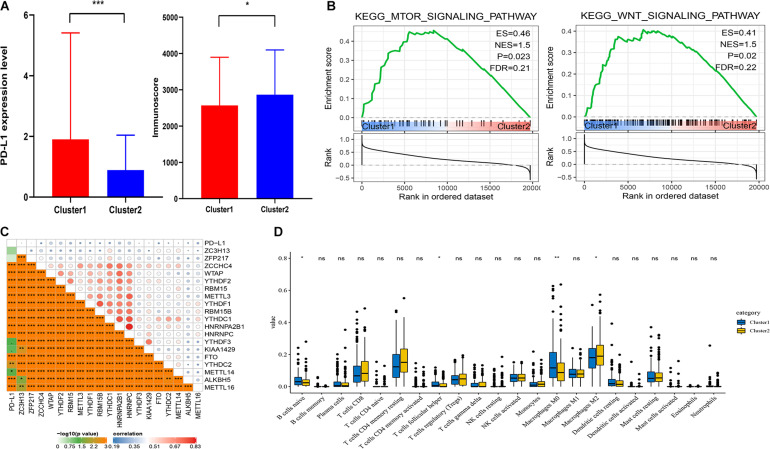
Association of PD-L1 and immune cell infiltration with m^6^A RNA methylation regulators. **(A)** Expression level of PD-L1 and immunoscore in cluster1 and cluster2. **(B)** GSEA showed that mTOR and Wnt signaling pathways were enriched in cluster1. **(C)** Correction between PD-L1 and m^6^A RNA regulators. **(D)** Expression levels of 22 immune cell infiltration in cluster1 and cluster2. *p < 0.05, **p < 0.01, and ***p < 0.001.

It showed that the expression of PD-L1 was dramatically higher in cluster1 (Figure 3A). Furthermore, PD-L1 was positively correlated with ZCCHC4, WTAP, YTHDF2, RBM15, METTL3, YTHDF1, RBM15B, YTHDC1, HNRNPA2B1, HNRNPC, KIAA1429, ALKBH5, and METTL16 (*p* < 0.001, Figure 3C).

### Association of Distinct Immune Cell Infiltration With m^6^A RNA Methylation Regulators

To analyze the correction between m^6^A regulators and TIME in HCC, we analyzed the immunoscore and immune infiltrate level of two subgroups (Figure 3A). The immunoscore was higher in cluster2 with a longer prognosis (*p* = 0.0265). Then we analyzed infiltration levels of 22 immune cell types between cluster1 and cluster2 (Figure 3D). The finding indicated that the infiltration levels of naïve B cells, T follicular helper cells, and macrophages M0 were higher in cluster1 (*p* < 0.05), whereas cluster2 showed higher infiltration levels of macrophages M2 (*p* < 0.05). To elucidate the underlying regulatory mechanisms that lead to temporal differences between cluster1 and cluster2, GSEA was used. Finally, the results indicated that mTOR and Wnt signaling pathways were correlated with cluster1 (Figure 3B).

### Accurate Prognostic Prediction of Signatures for m^6^A RNA Methylation Regulators

**FIGURE 4:**
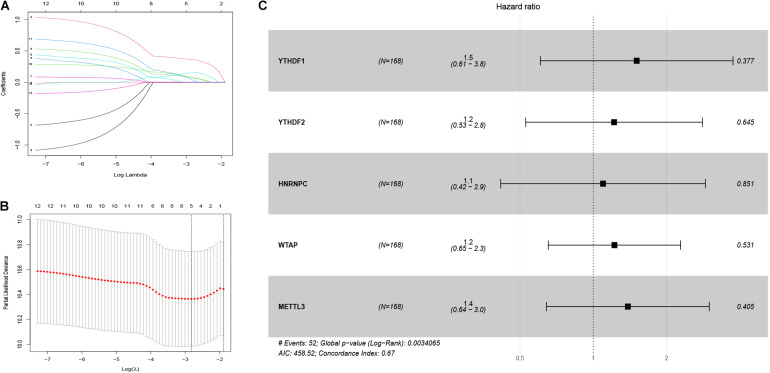
Identification of five m^6^A regulator-based risk signatures. **(A,B)** LASSO analysis of m^6^A regulator-based risk signatures. **(C)** Multivariate Cox analysis of the five m^6^A regulator-based risk signatures. ^#^The five regulators have strong predictive ability and the results has statistical significance.

**FIGURE 5:**
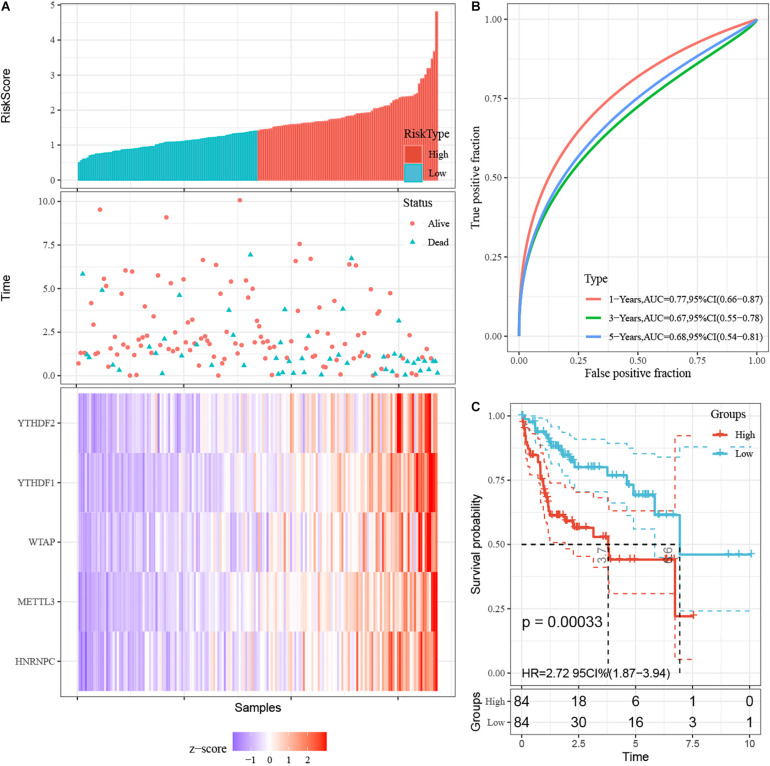
Construction of prognostic signatures for m^6^A regulator-based risk signatures. **(A)** Association of status and five m^6^A RNA regulators with risk score. **(B)** The area under AUC of five risk signatures in training dataset. **(C)** OS of high-risk group and low-risk group in training dataset.

First, 340 HCC patients were randomly divided into validation dataset (170 patients) and training dataset (170 patients). Second, in the training dataset, 13 m^6^A regulators were selected by using univariate regression analysis. Then five m^6^A regulators were identified based on the LASSO regression analysis, including YTHDF1, YTHDF2, HNRNPC, WTAP, and METTL3 (Figure 4). Subsequently, these candidate m^6^A regulators integrated into a predictive signature based on their risk coefficients. The formula went as follows: Risk Score = (0.4111 × YTHDF1 Expression) + (0.1969 × YTHDF2 Expression) + (0.0930 × HNRNPC Expression) + (0.2004 × WTAP Expression) + (0.3277 × METTL3 Expression). Afterward, according to the median risk score, patients were divided into high- and low-risk groups. The distributions of five m^6^A regulators’ expression profiles are shown in Figure 5A. The heatmap revealed higher expression levels of these m^6^A regulators in the high-risk group compared with the low-risk group (Figure 5A). The high-risk group had worse prognosis, compared with the low-risk group, based on Kaplan–Meier curve analysis (Figure 5C). The results were validated in the validation dataset (Supplementary Figure 3C). Moreover, we constructed a time-dependent ROC curve (AUC). As shown in Figure 5B, the AUC of five risk signatures was 0.77, 0.67, and 0.68 at 1, 3, and 5 years. Regarding the validation dataset, the 1-, 3-, and 5-year AUC values were 0.7, 0.63, and 0.55 (Supplementary Figure 3B). The results revealed that five risk signatures had a strong predictive ability in the prognosis of HCC.

### Risk Scores Was Associated With Clinical Features in HCC

**FIGURE 6:**
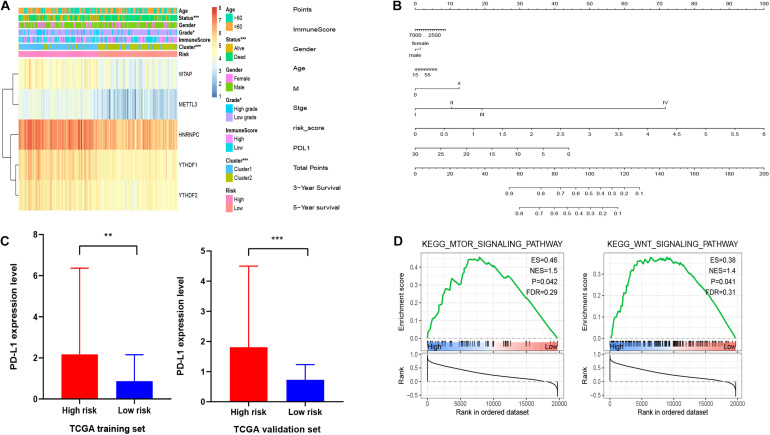
Association of clinical features and functional features with m^6^A regulator-based risk signatures. **(A)** Correction between clinical features and five m^6^A regulator-based risk signatures. **(B)** A nomogram for HCC patients. **(C)** Expression level of PD-L1 in high-risk and low-risk groups. **(D)** GSEA showed that mTOR and Wnt signaling pathways were enriched in high-risk group. *p < 0.05, **p < 0.01, and ***p < 0.001.

Furthermore, we aimed to explore the correction between risk score and clinical characteristics in the training dataset (Figure 6A). The heatmap showed that the high-risk group mainly contained cluster1 (*p* < 0.001), alive status (*p* < 0.001), and high-grade patients (*p* < 0.05). Then we found that YTHDF1, YTHDF2, HNRNPC, WTAP, and METTL3 had higher expression in high-risk group. Also, PD-L1 was expressed higher in high-risk group with worse OS, which was validated in the validation dataset (Figure 6C). Similarly, mTOR and Wnt signaling pathways were enriched in the high-risk group (Figure 6D). Then we built a nomogram for HCC patients to investigate the probability of 3- and 5-year OS. The results demonstrated the risk score could be a prognostic biomarker for HCC patients (Figure 6B). Finally, we found that the C-index was 0.738 and the calibration curve was close to the ideal curve, which indicated that the nomogram has good predictive effects (Supplementary Figure 4).

### Relationship Between Genetic Mutations of the m^6^A Regulator Signatures and Immune Cell Infiltration

**FIGURE 7:**
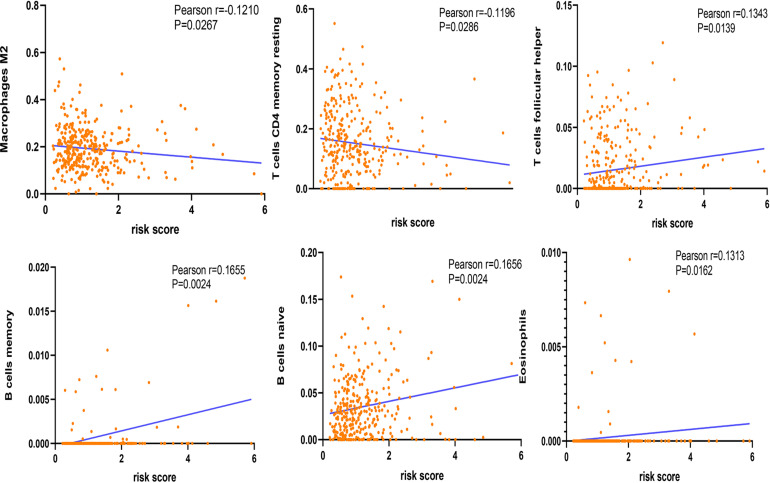
Correction between immune cells and risk score.

**FIGURE 8:**
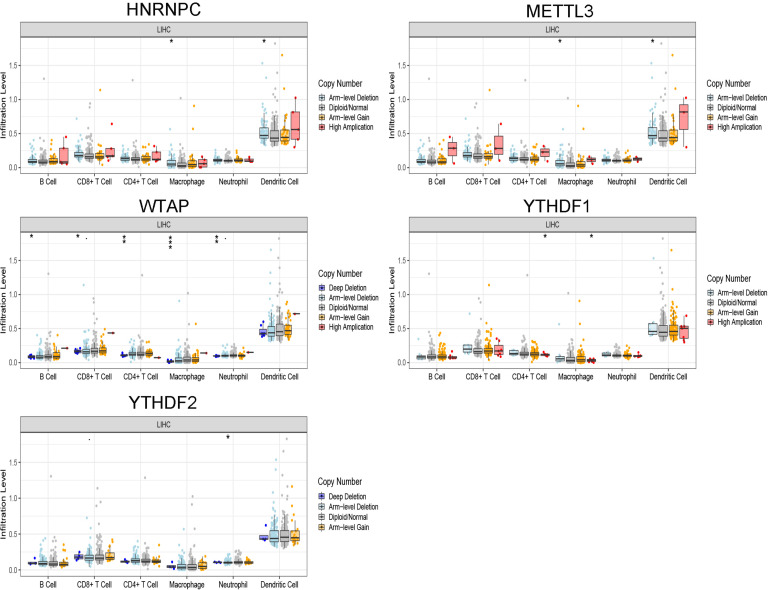
The CNAs of m^6^A regulatory factor signaling. *p < 0.05, **p < 0.01, and ***p < 0.001.

The correlation between risk score and immune cell infiltration was further analyzed (Figure 7). The risk score had a negative correction with infiltration levels of macrophages M2 and resting memory CD4 T cells (*p* < 0.05). Then the risk score was significantly corrected with B-cell memory, naïve B cells, T follicular helper cells, and eosinophils (*p* < 0.05). The results confirmed that the risk signatures based on m^6^A regulators were related to the HCC immune microenvironment. Moreover, to clarify the potential mechanism of risk score and different immune cell infiltration, the effect of somatic CNA based on m^6^A modulator signal on immune cell infiltration was analyzed (Figure 8). The CNAs of m^6^A regulatory factor signaling, mainly including deep deletion and arm-level deletion, could affect the infiltration levels of B cells, CD8^+^ T cells, CD4^+^ T cells, neutrophils, dendritic cells, and especially macrophages (*p* < 0.05). It revealed that the five m^6^A regulators play an important role in TIME of HCC patients.

## Discussion

The m^6^A regulates gene expression, which regulates cellular processes such as cell self-proliferation, differentiation, invasion, and apoptosis (He et al., 2019). The m^6^A is immobilized by m^6^A methyltransferase, removed by m^6^A demethylases, and recognized by the reader proteins to regulate RNA metabolism and progression of various tumors (Li et al., 2017; Ma et al., 2017; Roignant and Soller, 2017). However, the role of some m^6^A regulators in tumor is unclear. For example, METTL14 plays different roles in different types of tumors. Yang et al. (2020) showed that METTL14 inhibits the proliferation and metastasis of colorectal cancer by downregulating the oncogenic long non-coding RNA XIST. Wang M. et al. (2020) reported that upregulation of METTL14 promotes the growth and metastasis of pancreatic cancer by mediating the increase of PERP mRNA *N*^6^-adenosine methylation. Therefore, the expression levels and functions of m^6^A regulators are complex in different tumors. The mechanism of RNA methylation in tumors needs to be further investigated. Currently, the effect of m^6^A RNA methylation in the TIME of HCC needs to be analyzed further.

In the research, we demonstrated the expression of m^6^A regulators in HCC, its prognostic value, and the effect of TIME, YTHDF1, YTHDF2, HNRNPC, WTAP, and METTL3. METTL3 and HNRNPC dramatically decreased in HCC compared with normal tissues (*p* < 0.001). YTHDF2 (*p* < 0.001) and WTAP (*p* < 0.05) were significantly upregulated in HCC tissues. However, the expression level of YTHDF1 was down-regulated in HCC tissues. Then, two subtypes of HCC, cluster1 and cluster2, were identified. We found that all m^6^A regulators upregulated in cluster1. Also, OS and PFS of patients in cluster1 were worse compared with cluster2. Compared with cluster2, cluster1 was closely associated with lower immune score and higher PD-L1 expression level. The results were confirmed by a previous report, which showed that patients with high PD-L1 expression had a distinct poorer prognosis than those with low PD-L1 expression (Gao et al., 2009). Moreover, the expression levels of immune cells were significantly deferent in the two subtypes. The analysis showed that the infiltration levels of naïve B cells, T follicular helper cells, and macrophages M0 were higher in cluster1, whereas the level of macrophages M2 was lower. The results showed that the m^6^A regulators were closely associated with prognosis and TIME in HCC patients. The GSEA reveled that the functional characteristics of HCC, especially Wnt and mTOR signaling pathways, were mainly enriched in cluster1. Tang et al. (2020) reported that m^6^A demethylase inhibits tumor by mediating Wnt signaling. Zhao et al. (2020) reported that m^6^A RNA modification regulates mTOR signal pathway in gastrointestinal cancer. Also, Zhang H. et al. (2020) found that the m^6^A regulator METTL3 promotes the progression of retinoblastoma through mTOR signal pathway. The findings revealed that m^6^A regulators could affect the progression of HCC by targeting Wnt and mTOR pathways, which could provide a new therapeutic strategy for the treatment of HCC.

Furthermore, high- and low-risk group patients were identified based on the five m^6^A regulator-based risk signatures. Interestingly, we found that cluster1 was distinctly enriched in the high-risk group. Similarly, high-risk group with high PD-L1 expression level had worse prognosis than low-risk group with low PD-L1 expression level in the training dataset and the validation dataset. The nomogram indicated that the risk score could effectively predict the prognosis of patients with HCC. Regarding the five m^6^A regulator-based risk signatures, four of five m^6^A regulators, including YTHDF1, YTHDF2, WTAP, and METTL3, could facilitate the progression of HCC, and the results were confirmed by previous reports (Chen et al., 2018, 2019; Liu et al., 2020; Zhang C. et al., 2020). However, Zhong et al. (2019) showed that YTHDF2 suppresses cell proliferation and growth in HCC. Moreover, five m^6^A regulators are also associated with different cancers. For example, YTHDF1, YTHDF2, and especially METTL3 are associated with gastric cancer. YTHDF1 and its m^6^A-mediated regulation of Wnt/β-catenin signaling promote gastric cancer progression. METTL3-mediated m^6^A modification facilitates gastric cancer progression and has poor prognosis (Pi et al., 2020; Shen et al., 2020; Wang Q. et al., 2020). Also, HNRNPC and YTHDF1 have an effect on prognosis in breast cancer patients (Wu et al., 2018; Anita et al., 2020). Also, the results were confirmed by the data obtained from Kaplan–Meier Plotter (Supplementary Figure 5). The results showed that the dysregulation of specific m^6^A regulators plays a key role in the progression of various kinds of tumors.

Tumor microenvironment is involved in the regulation of patient prognosis and response to treatment (Fridman et al., 2012; Galon et al., 2014; Hui and Chen, 2015). A previous study showed that tumor-infiltrating lymphocytes could serve as prognostic biomarkers and targets for immunotherapy in HCC (Ding et al., 2018). However, the mechanism of immune infiltration in response to HCC is largely unclear. In this study, we analyzed the correction between immune cell infiltration and risk score. We found that the risk score was positively associated with the infiltration levels of memory B cells, naïve B cells, T follicular helper cells, and eosinophils, and negatively associated with the infiltration levels of macrophages M2 and resting memory CD4 T cells. Zheng et al. (2020) reported that the RNA m^6^A methylation and its reader proteins play a key regulatory role in early B-cell development. These findings reveled that the m^6^A regulators are associated with TIME in HCC to some extent. Furthermore, we found that the CNAs of regulators were closely related to the immune infiltration levels, including B cells, CD8^+^ T cells, CD4^+^ T cells, neutrophils, dendritic cells, and especially macrophages. It is further confirmed that the m^6^A regulators could affect TIME regulation in HCC.

However, there are some limitations in this research. First, the data of research are only obtained from the TCGA datasets. We lack our own independent clinical sample data to verify our findings. Then the results of our research need further experimental verification. In the future, we will further research the correction between m^6^A regulators and TIME in HCC.

In summary, we systematically assessed the relationship of TIME, PD-L1, and m^6^A regulators in HCC. According to the expression levels of the m^6^A regulators, HCC patients were divided into cluster1 and cluster2. The two clusters were significantly different in PD-L1 expression level, immunoscore, prognosis, and TIME in HCC. HCC patients with higher PD-L1 expression or immunoscore were associated with worse prognosis. Then high-risk group and low-risk group patients were identified based on the five risk signatures. The high-risk group was significantly associated with higher PD-L1 expression levels, higher grades, and worse OS. The GSEA results revealed that the m^6^A regulators were associated with the malignant functional features of HCC, including Wnt and mTOR signaling pathways. Therefore, the m^6^A regulators are associated with TIME in HCC, which could provide a new treatment strategy for HCC patients.

## Data Availability Statement

The datasets presented in this study can be found in online repositories. The names of the repository/repositories and accession number(s) can be found in the article/Supplementary Material.

## Author Contributions

XX: conceptualization, methodology, writing-review, and editing. YX: methodology, resources, software, formal analysis, and writing-original draft. XH: methodology, resources, formal analysis, and writing-original draft. JD: methodology, resources, software, and writing-original draft. LX: validation and data curation. YL and XZ: visualization and supervision. WC and XL: visualization. All authors contributed to the article and approved the submitted version.

## Conflict of Interest

The authors declare that the research was conducted in the absence of any commercial or financial relationships that could be construed as a potential conflict of interest.

## Footnotes

1 https://portal.gdc.cancer.gov/

2 http://www.bioconductor.org/

3 http://sangerbox.com/

4 https://cibersort.stanford.edu/

5 https://www.r-project.org/

## Supplementary Material

Supplementary Figure 1 Flowchart of the article.

Supplementary Figure 2 Correlation of consensus clustering for m^6^A RNA methylation regulators. Consensus clustering matrix from *k* = 2–9.

Supplementary Figure 3 Construction of prognostic signatures for m^6^A regulator-based risk signatures in the validation dataset. **(A)** Association of status and five m^6^A RNA regulators with risk score. **(B)** The area under AUC of five risk signatures. **(C)** OS of high-risk group and low-risk group.

Supplementary Figure 4 The calibration curve of 3 and 5 years.

Supplementary Figure 5 OS of YTHTF1, METTL3, and HNRNPC in gastric carcinoma.
