# Supplementary figures and images for: Comprehensive Analysis of the Immune Infiltrates and PD-L1 of m6A RNA Methylation Regulators in Hepatocellular Carcinoma
Source: Front Cell Dev Biol. 2021 Jun 30;9:681745. doi: 10.3389/fcell.2021.681745 (PMC8277965; doi:10.3389/fcell.2021.681745)

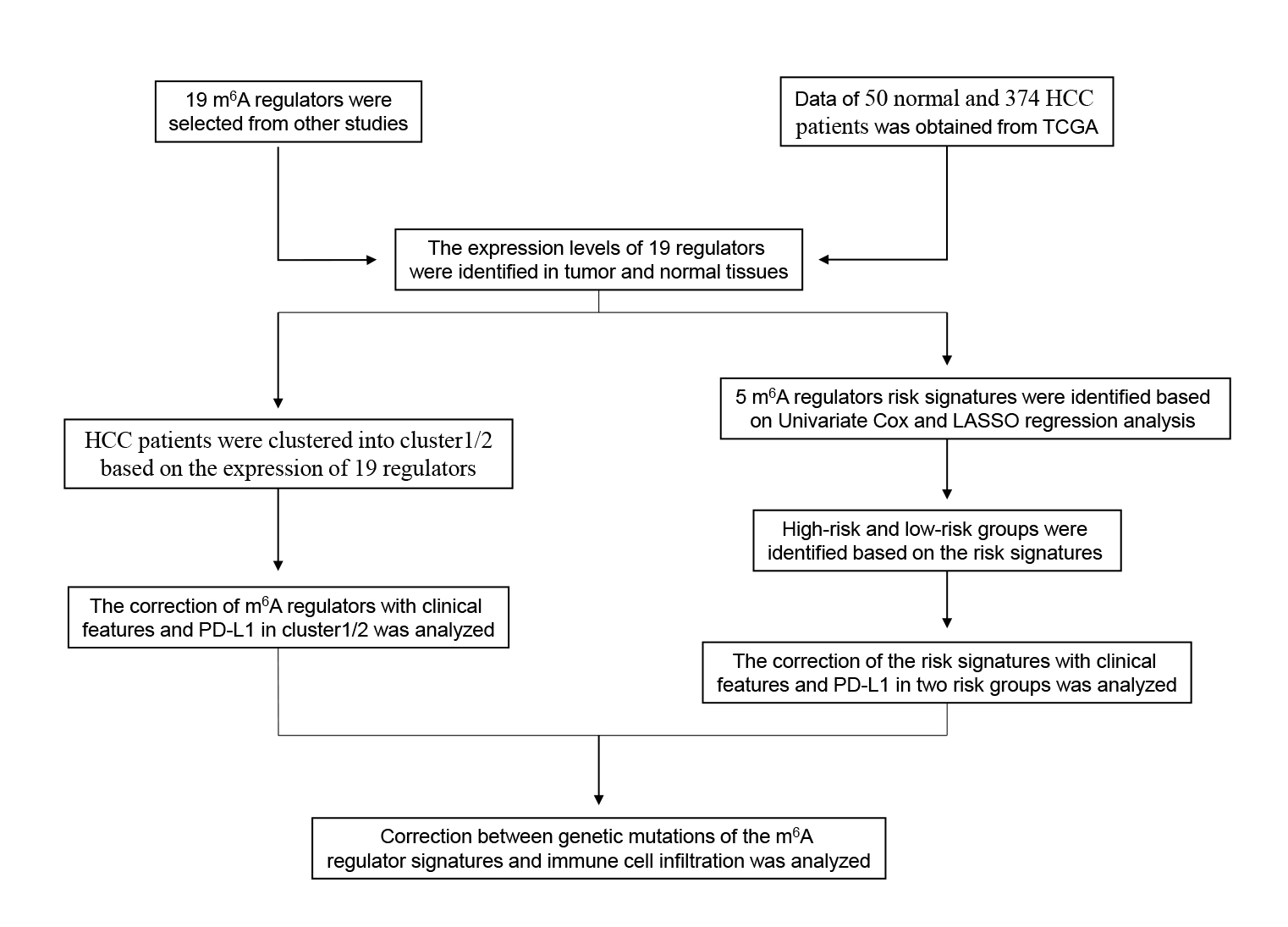

Supplement: Supplementary Figure 1 — Flowchart of the article. [file Image_1.JPEG]

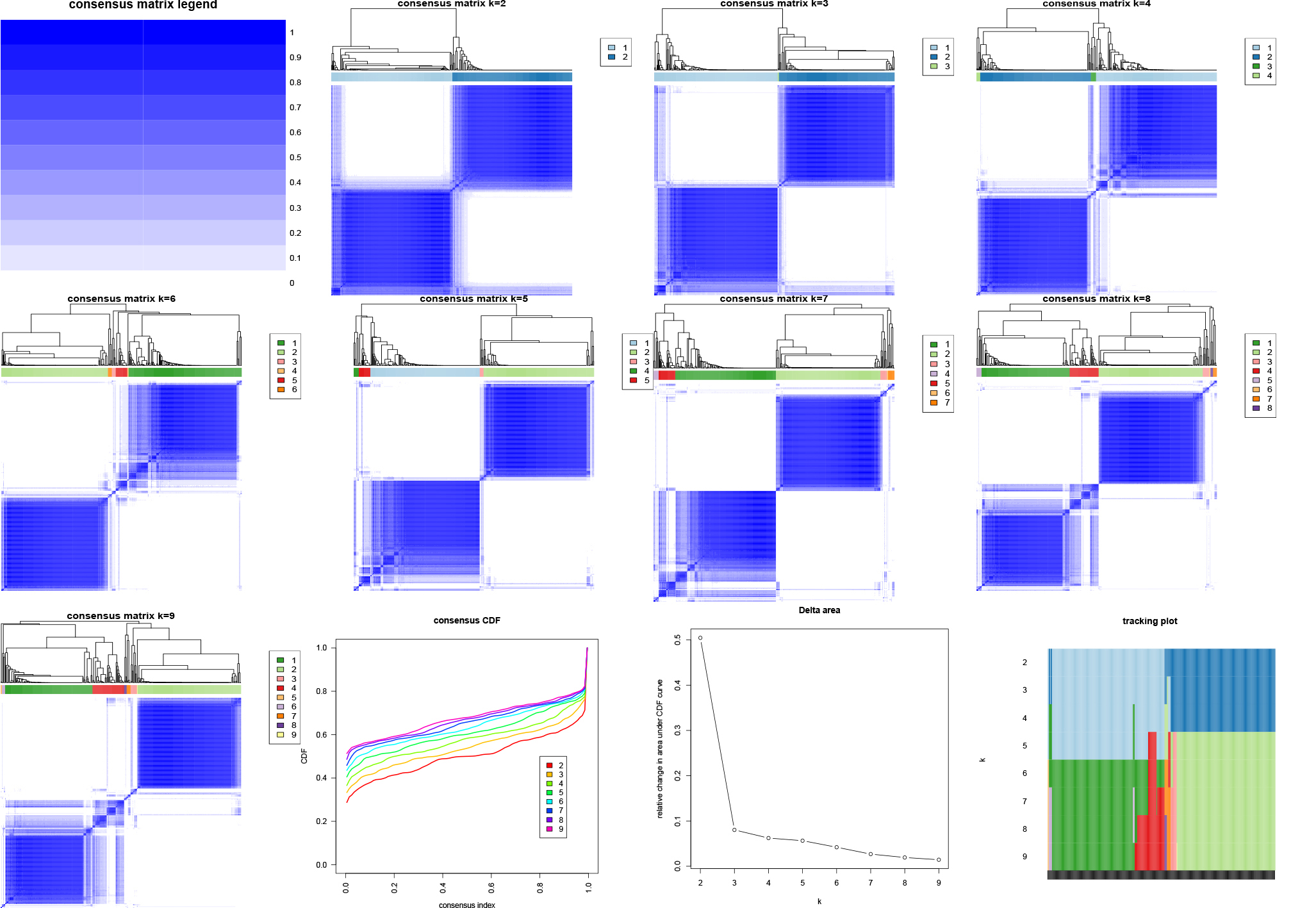

Supplement: Supplementary Figure 2 — Correlation of consensus clustering for m6A RNA methylation regulators. Consensus clustering matrix from k = 2–9. [file Image_2.JPEG]

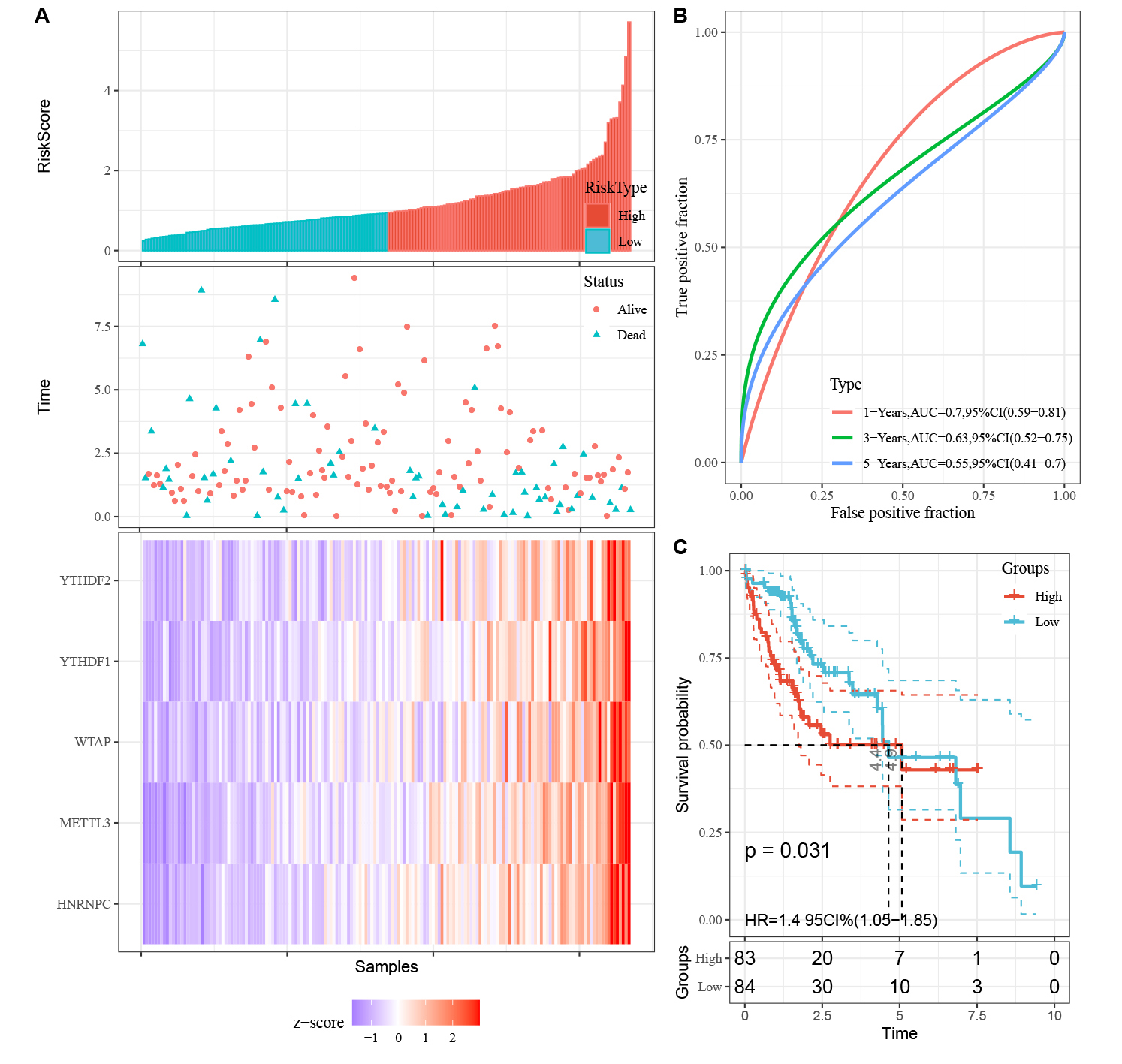

Supplement: Supplementary Figure 3 — Construction of prognostic signatures for m6A regulator-based risk signatures in the validation dataset. (A) Association of status and five m6A RNA regulators with risk score. (B) The area under AUC of five risk signatures. (C) OS of high-risk group and low-risk group. [file Image_3.JPEG]

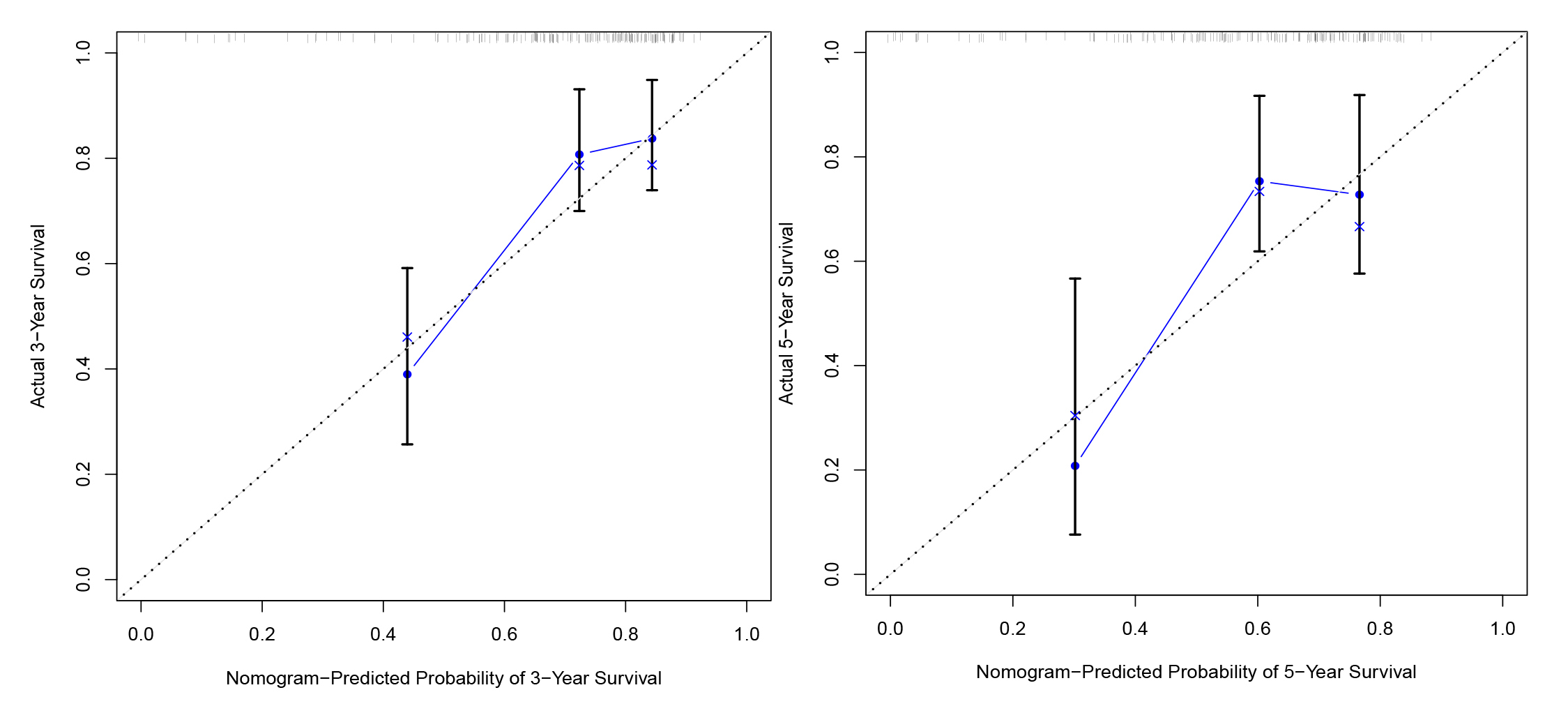

Supplement: Supplementary Figure 4 — The calibration curve of 3 and 5 years. [file Image_4.JPEG]

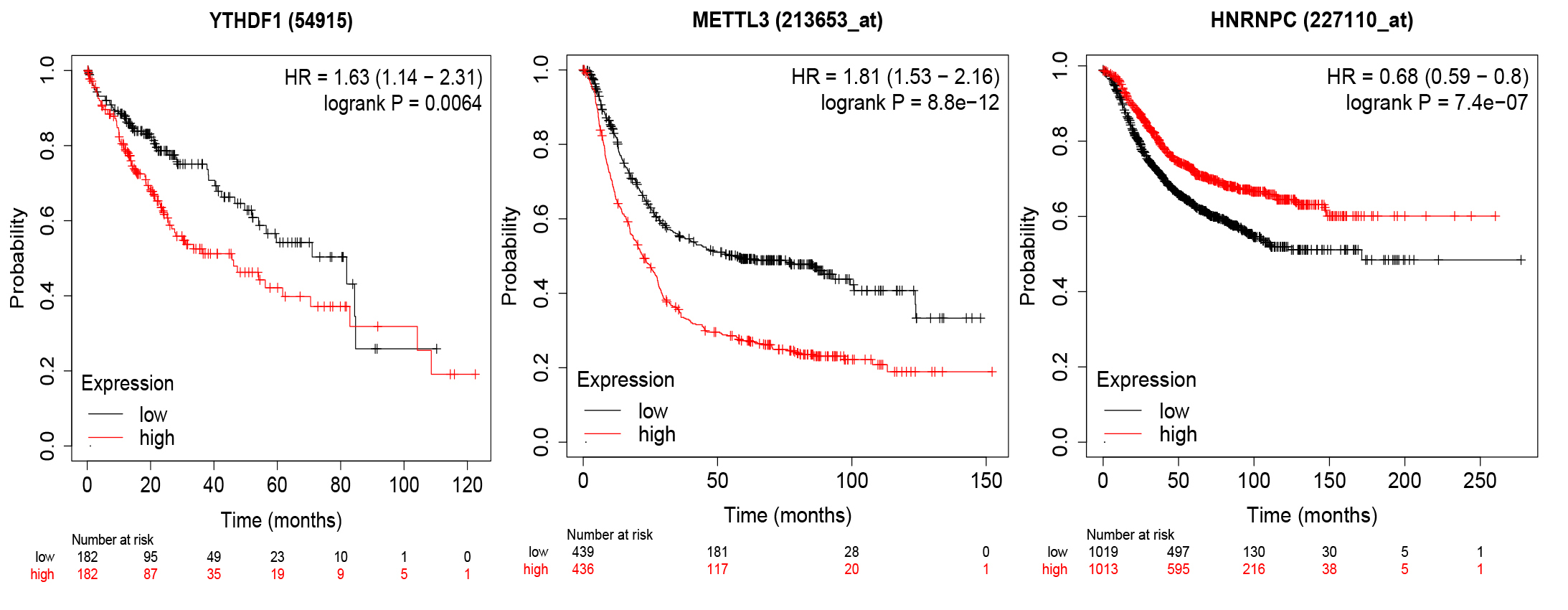

Supplement: Supplementary Figure 5 — OS of YTHTF1, METTL3, and HNRNPC in gastric carcinoma. [file Image_5.JPEG]
